# Controlled peritoneal drainage improves survival in children with abdominal compartment syndrome

**DOI:** 10.1186/s13052-015-0134-6

**Published:** 2015-04-08

**Authors:** Yu-Jian Liang, Hui-min Huang, Hong-ling Yang, Ling-ling Xu, Li-dan Zhang, Su-ping Li, Wen Tang

**Affiliations:** Department of Pediatric Intensive Care Unit, First Affiliated Hospital, Sun Yat-sen University, 58 Zhongshan Second Road, Guangzhou, Guangdong 510080 P R China; Department of Laboratory, Guangzhou Women and Children’s Medical Centre, Guangzhou Medical College, Guangzhou, Guangdong China

**Keywords:** Percutaneous catheter decompression, Abdominal compartment syndrome, Intra-abdominal pressure, Pediatric intensive care unit (PICU), Children, Survival

## Abstract

**Background:**

Children with massive ascites can develop abdominal compartment syndrome (ACS), which has been identified as an independent risk factor for mortality.

**Objectives:**

The objective of this study was to assess the effectiveness of volume-controlled percutaneous catheter drainage (PCD) for treating children with massive ascites and ACS.

**Methods:**

A retrospective descriptive study was conducted; Comprising 12patients with ACS with massive ascites treated with volume-controlled PCD in a pediatric intensive care unitof a university hospital in southern China from April 2011 to June 2013.

**Results:**

The etiology of ascites in these children included abdominal tumor (8/12), capillary leak after liver or kidney transplantation (2/12) and urine leakage (2/12). Intra-abdominal hypertension was closely associated with multiple organ dysfunction and high mortality. Digestive and pulmonary functions were the most frequently affected by ACS, while the cerebrum was the least involved. Treatment with ultrasound-guided PCD significantly decreased intra-abdominal pressure, abdominal circumference, and indices of organ dysfunction. PCD treatment also significantly improved glomerular filtration rate and PaO_2_/FiO_2_. Complications of PCD included abdominal infection (1/12) and electrolyte imbalance (4/12). The mortality rate of patients treated with PCD was 25%, which was lower than previous reports.

**Conclusions:**

Controlled peritoneal drainage is a minimally invasive and safe decompression method that is effective in patients with ACS, and should be considered in children with massive ascites.

## Introduction

Intra-abdominal hypertension (IAH), occurs in 50% or more of critically ill patients, and is an identified independent risk factor for mortality [1]. IAP can lead to abdominal compartment syndrome (ACS), which is closely associated with dysfunctions of the cerebrum and the digestive, respiratory, cardiovascular, and renal systems, as well as significantly increased mortality [2]. Awareness of ACS has grown in the past decade, and mortality due to ACS in adults has decreased from 60% to 37% [3-5]. However, the mortality rate due to ACS in children has remained stable at 40% to 60% [6,7]. This high mortality is partially attributable to poor recognition of ACS in pediatric medicine. A recent survey showed that only 47% of pediatricians could correctly identify ACS in children, 24% had never measured IAP, and only half (51%) had treated a child with ACS [8]. Similarly, there have been too few reports of ACS in the pediatric intensive care unit (PICU).

Treatments of ACS include medical and surgical management. Selecting an effective therapeutic intervention for ACS is very important. Treatments for ACS in children, as in adults, include improving abdominal wall compliance, evacuating intraluminal contents, and maintaining APP through medical management. Prokinetic agents, diuretics, and sedatives are recommended. Surgical intervention, such as decompressive laparotomy or temporary abdominal closure, has been suggested and is usually performed when IAP reaches more than 20 mmHg or a progressive organ dysfunction is present [9-11]. However, surgical complications, such as enteric fistulae and chronic incisional hernia, can be considerable [12-14].

Recently, percutaneous catheter drainage (PCD), a minimally invasive therapy, has been recommended to replace traditional surgical interventions in the patient with intra-peritoneal fluid or blood secondary to ACS [15,16], and for patients with massive ascites. Some case studies involving children have been reported [17,18], but not a case–control analysis or clinical trials report. Furthermore, most reports were limited to children with ACS due only to burns and trauma.

This is a retrospective descriptive study of the effectiveness of PCD in children with ACS. The primary goal of this study was to investigate the effectiveness and safety of PCD in children with ACS due to factors other than burns or trauma, and to report physiological changes and mortality in patients undergoing this procedure, relative to supportive care.

## Materials and methods

This retrospective study included children who were diagnosed with ACS with massive ascites and were admitted to the PICU of First Affiliated Hospital of Sun Yat-sen University, a tertiary care university hospital in southern China, from April 2011 to June 2013. The Ethics Committee of First Affiliated Hospital of Sun Yat-sen University, China, waived the need for approval since the study included no modifications to standards of ACS treatment.

According to the consensus definition adopted in 2013 by the World Society of the Abdominal Compartment Syndrome (WSACS), IAH is defined as IAP > 10 mmHg, and is divided into 4 grades, based on mmHg of IAP: Grade I, 10–15 mmHg; GradeII, 16–20 mmHg; GradeIII, 21–25 mmHg; and GradeIV, >25 mmHg. ACS in children is defined as IAP > 10 mmHg with evidence of new organ dysfunction or failure [19].

IAP was measured indirectly by examining intravesical pressure via a Foley catheter, in accordance with the standard procedure [14]. Briefly, the patient was placed supine, and the correct placement of the Foley catheter in the bladder was verified. A needle connected to the Foley tube is inserted, and the Foley catheter was transiently clamped. Sterile saline was injected into the empty bladder (1 mL/kg for small children and up to a maximum of 25 mL for older children). The IAP was measured 30–60 s after installation and at the end-expiration; the midaxillary line at the iliac crest was taken as the zero reference. The Foley catheter was unclamped, and the above steps were repeated, when necessary.

Between April 2011 and June 2013, 20 patients with massive ascites met the ACS criteria. Twelve were treated with PCD for inclusion in this study. Patients’ demographics, diagnoses, IAP, mean arterial pressure (MAP), oxygenation indices (ratio of partial pressure arterial oxygen [PaO_2_] to fraction of inspired oxygen [FiO_2_], or PaO_2_/FiO2), partial pressure of carbon dioxide (PCO2), serum creatinine, 24-hour urine volumes, drainage time, Glasgow Coma Score (GCS) [20], Pediatric Risk of Mortality III (PRISM III) score [21], number of dysfunctional organs, mortality and survival time were recorded in a computerized database. Organ dysfunction was determined based on definitions for pediatric organ dysfunction in sepsis [22] and Kidney Disease Improving Global Outcomes (KDIGO) for diagnosing acute kidney injury [23]. IAP, abdominal circumference, organ function, GCS and PRISM III score was also recorded before and after PCD to observe the effect on prognosis.

After a diagnosis of ACS attributable to massive ascites was determined, we firstly used medical management. All the patients were treated with gastrointestinal decompression, rectal enemas, prokinetic medications, diuretics, adequate sedation, and analgesia, as required. If the IAP did not decrease or became higher after 24 hours, we performed bedside PCD with ultrasonographic localization, after the patients’ parents consented to the procedure. We used a deep-venous catheter or pigtail catheter for drainage. A valve opening into the drain bag was used to control flow rate. The flow rate was set to 5–10 mL/kg body weight per hour throughout the procedure. Fluid output was typically ~30 mL/kg body weight during the first day. When hemodynamic parameters were stable, the drainage output was increased to 100–200 mL/day. The goal of the procedure was to reduce IAP at a rate of 2–3 mmHg/d. When the fluid output reached <20 mL/d and the abdominal distension disappeared, the drainage tube was turned off. Every 2 hours, the doctors in charge adjusted and documented the flow rate and volume of ascites drainage, based on the IAP and hemodynamic parameters. If IAP remained normal 24 hours after drainage was stopped, the catheter was removed to complete the PCD procedure.

All data were prepared and compiled for statistical analyses using SPSS (version 13.0 for Windows). Descriptive statistics were analyzed by independent samples *t*-test or the rank sum test. Categorical data were compared with Fisher’s exact test. All tests were one-tailed, and *P* < 0.05 was considered statistically significant.

## Results

**Figure 1:**
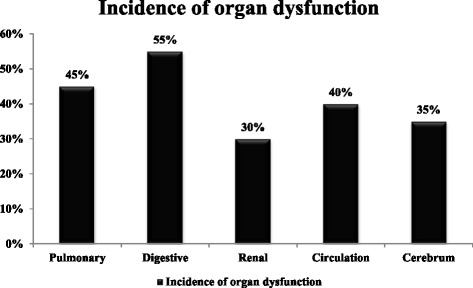
**Incidence of organ dysfunction in ACS child patients who received PCD.** The digestive and respiratory systems were the most frequently involved, whereas central nervous system dysfunction was least often observed.

**Figure 2:**
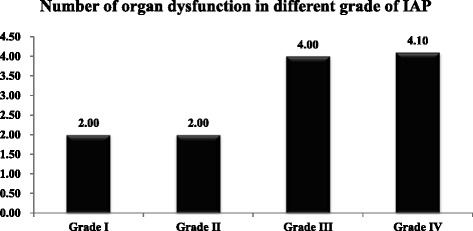
**The number of dysfunctional organs closely and positively correlated with the grade of IAP.**

Included in this study were 12 children with PCD treated between April 2011 and June 2013; 7 boys and 5 girls, median age 2.7 years (range 0.17-11 years). The significant effects of ACS on organ dysfunction were manifested in the digestive and respiratory systems, heart, kidney, and cerebrum (Figure 1). The digestive and respiratory systems were the most frequently involved, whereas dysfunction of the central nervous system was least often observed. Furthermore, the number of dysfunctional organs closely correlated with the grade of IAP (Figure 2).

In the patients who received PCD treatment, the IAP, abdominal circumference, serum creatinine, gastric retention, gastrointestinal bleeding, and organ dysfunction all significantly improved; urine output and glomerular filtration rate also improved (Table 1). The mortality was 25%.

**Table 1:** **Clinical parameters before and after PCD**

	**Before PCD**	**After PCD**	***P***
IAP, mmHg	24.2 ± 8.46	12.2 ± 8.40	0.001
Abdominal circumference, cm	59.08 ± 12.99	54.83 ± 14.74	<0.001
PaCO_2_, mmHg	41.33 ± 11.26	38.25 ± 4.86	0.43
Serum creatinine, μmol/L	49.64 ± 32.45	25.73 ± 20.32	0.001
Urine output, mL · kg^−1^ · h^−1^	2.11 ± 1.09	3.17 ± 1.22	0.04
Glomerular filtration rate	55.04 ± 55.19	104.40 ± 109.38	0.003
Gastric retention	83%(10/12)	33%(4/12)	0.04
Gastrointestinal bleeding	75%(9/12)	17%(2/12)	0.01
MAP, mmHg	78.63 ± 16.90	64.69 ± 40.26*	0.53
PaO_2_/FiO_2_	189.42 ± 75.01	228.08 ± 140.07*	0.37
Number of dysfunctional organs	3.08 ± 1.31	1.5 ± 1.6*	0.01
Glasgow coma score	10.67 ± 3.87	11.25 ± 5.15*	0.78
PRISM III	20.00 ± 5.72	28.33 ± 31.49*	0.37

*Three of 12 patients died during PCD treatment. Their MAP, PaO_2_/FiO_2_, number of dysfunctional organs, PRISM III score and Glasgow coma score were recorded as zero or the lowest.

The etiology of ascites in these children included abdominal tumor (8/12), capillary leak after liver or kidney transplantation (2/12) and urine leakage (2/12). The complication rates related to PCD management included electrolyte imbalance (4/12) and abdominal infection (1/12). When using central venous catheters for PCD, the incidence of blockage was 83.3% (5/6), however, blockage did not occur with pigtail catheters (Table 2).

**Table 2:** **Diagnosis, drainage, and prognosis of patients given PCD**

**Patient**	**Main diagnosis**	**IAP, mmHg**	**Catheter type**	**Drain time (day)**	**Blockage** ^**a**^	**Electrolyte imbalance** ^**b**^	**Abdominal infection** ^**c**^	**Thirty-day prognosis**
1	Kidney transplantation	19	Abdominal drain tube	11	–	–	–	Survival
2	Liver transplantation	30	Abdominal drain tube	6	–	–	–	Death
3	Hepatoblastoma rupture	24	CVC^d^	10	–	+	–	Survival
4	Hepatoblastoma rupture	17	CVC	3	+	–	–	Death
5	Adrenal gland neoplasm rupture	34	CVC	3	+	–	–	Survival
6	Urine leakage	21	CVC	23	+	–	–	Survival
7	Abdominal rupture	24	CVC	6	+	+	–	Survival
8	Hepatoblastoma rupture	40	CVC	20	+	–	–	Survival
9	Abdominal rupture	14	Pigtail catheter	8	–	–	+	Survival
10	Urine leakage	30	Pigtail catheter	8	–	–	–	Survival
11	Adrenal gland rupture	26	Pigtail catheter	3	–	+	–	Death
12	Adrenal gland neoplasm rupture	11	Abdominal drain tube	20	+	+	–	Survival

^a^Catheter blockage during PCD (+, yes; −, no).

^b^Electrolyte imbalance occurred as a complication of PCD during the drainage (+, yes; −, no).

^c^Abdominal infection as a complication of PCD during the drainage (+, yes; −, no).

^d^CVC, central venous catheter.

## Discussion

ACS-related mortality is high, according to various studies of children admitted to the PICU [24]. If ACS is not recognized and treated promptly, mortality can reach 90% to 100% [9]. In general, PCD is recommended for ACS, but there has been little work on its effectiveness in pediatric patients. Our present study is a retrospective clinical trial investigating the effectiveness and safety of PCD in children. The results indicated that PCD could increase the survival rate and prolong the survival time of children with ACS that is due to massive ascites from abdominal tumor and urine leakage. In patients who received PCD, the IAP was significantly decreased, and the mortality was lower than the previous reports of ACS. Although we cannot verify whether the difference in mortality was due to differences in etiology of ACS, we recommend that patients with massive ascites and ACS should be considered to receive PCD.

The safety of PCD has been seldom studied in the pediatric literature. In this report, abdominal infection and electrolyte imbalance occurred during the PCD treatment. However, these complications were not significant and could be easily managed. It is also noteworthy that catheter blockage may develop during PCD. When using central venous catheters for PCD, the incidence of blockage was 83.3% (5/6). To solve this problem, we used pigtail catheters rather than central venous catheters for drainage. The incidence of blockage reduced to zero. Based on our experience, we recommend using pigtail catheters for PCD treatment.

The timing of PCD has been rarely reported, and it is not clear from the published literature which clinical conditions necessitate PCD decompression. Cheatham and Safcsak [25] suggested that PCD should be performed in patients with moderate to severe IAP (>21-25 mmHg). From this study, we also observed that patients with IAP levels over Grade III had significantly more organdysfunction. Although in this study we cannot verify whether the organ dysfunctionwas due to ACS, we suggest that patients with IAH higher than Grade III (i.e. >21–25 mmHg) should be considered for PCD. Further investigation is needed to confirm this.

For patients with abdominal hemorrhage, hypovolemic shock attributed to PCD may occur when bloody ascites is drained too fast. However, hypovolemic shock did not occur in our study, perhaps due to the volume-control of daily drainage. In contrast, venous return volume and organ perfusion improved the decrease in IAP, which led to better physiological parameters and prolonged survival.

Patients with ACS have multiple organ dysfunctions. Our data showed that digestive and respiratory systems were the most frequently involved, whereas central nervous system dysfunction was least often observed. The pathophysiology of ACS is intricate and complex. Abdominal disease is the most common etiology of ACS, such as abdominal surgery, abdominal trauma, and ileus [26]. Therefore, abdominal viscera are the most commonly affected organs. Gastrointestinal mucosa is very sensitive to ischemia attributed to IAH. This leads to gastrointestinal bleeding and gastric retention. When IAP increases, the diaphragm is pushed up, and intra-pleural pressure increases; intra-thoracic volume and chest wall compliance is reduced, which leads to ventilation-perfusion mismatching, hypoxia, hypercarbia and respiratory acidosis.

In addition, IAH increases the compromise of multiple intra- and extra-abdominal organ systems [27]. Our study revealed that there was a significant association between the number of dysfunctional organs and bladder pressure (Figure 2). This may be related to the decrease of blood flow in intra-abdominal organs when IAP rise, which results in diminishing the flow of portal venous, hepatic and mesenteric arterial, renal plasma, and glomerular filtration rate. Because of far-reaching effects on both intra- and extra-abdominal organs, progressive elevation of IAP can lead to increase of organ dysfunction and, eventually, the death of the child patient. Therefore, the extent of IAH may be related to mortality. This needs to be studied further.

### Limitations

Since this study has a small sample size, the statistical power of the results is not great and it is difficult to conclude whether other confounding variables could account for mortality. Prospective randomized controlled and well-powered studies need to be designed to confirm or dispute our conclusions.

## Conclusions

Controlled peritoneal drainage is a minimally invasive, safe, and effective method of decompression that should be considered for pediatric patientswith massive ascites. Whenever technically available, volume-controlled PCD should be performed as early as possible to avoid the need for surgical decompression. Future prospective studies should be conducted to formulate evidence-based recommendations regarding the rate of decompression and amount of drainage for pediatric patients.

### Recommendation

Our study was limited by the small number of ACS patients, as well as its observational design. Prospective studies should be conducted to formulate evidence-based recommendations regarding the rate of decompression and amount of drainage for pediatric patients.

## Abbreviations

ACS: Abdominal compartment syndrome
APP: Abdominal perfusion pressure
IAP: Intra-abdominal pressure
MAP: Mean arterial pressure
KDIGO: Kidney Disease Improving Global Outcomes
PCD: Percutaneous catheter drainage
PICU: Pediatric intensive care unit
GCS: Glasgow Coma Score
PRISM III: Pediatric Risk of Mortality III
